# Government Trust and Motivational Factors on Health Protective Behaviors to Prevent COVID-19 Among Young Adults

**DOI:** 10.3389/ijph.2022.1604290

**Published:** 2022-04-13

**Authors:** Nicolás Bronfman, Paula Repetto, Pamela Cisternas, Javiera Castañeda, Paola Cordón

**Affiliations:** ^1^ Engineering Sciences Department, Universidad Andres Bello, Santiago, Chile; ^2^ Research Center for Integrated Disaster Risk Management ANID/FONDAP/15110017, Santiago, Chile; ^3^ Department of Psychology, Pontificia Universidad Católica de Chile, Santiago, Chile; ^4^ Industrial and Systems Engineering Department, Pontificia Universidad Católica de Chile, Santiago, Chile

**Keywords:** COVID–19, protective behaviors, worry, subjective norms, government trust

## Abstract

**Objective:** The purpose of this study was to determine the influence of government trust on young adults’ adoption of health behaviors to prevent infection with the SARS-CoV-2 virus.

**Method:** We tested the hypothesis that government trust would directly and indirectly (through worry/fear and subjective norms) influence the adoption of health-protective behaviors. A sample of 1,136 university students completed a web survey after Chile’s first wave of infections.

**Results:** The results indicate that low government trust only indirectly (through subjective norms) influenced health-protective behaviors. Conversely, worry/fear was the primary motivating factor for adopting health-protective behaviors in young adults, followed by subjective norms.

**Conclusion:** In scenarios where people perceive low government trust, emotions and social norms are the motivational factors with the most significant predictive power on the adoption of health-protective behaviors.

## Introduction

Human behavior in a pandemic significantly affects the transmission of the virus that produces the disease. Therefore, it is essential to encourage people to adopt the authorities’ suggested protective measures to manage the crisis successfully [1]. Several factors determine the intention and the adoption of protective measures in a pandemic. Among these factors, the most important correspond to motivational factors such as trust in authorities, attitudes, subjective norms, fear, and worry [2–6]; as well as psychosocial conditions such as anxiety and depression [5, 7, 8]; socio-demographic factors [9–11]; among others. For the motivational factors, trust in the authorities plays an essential role in managing a pandemic [12–14] since it is crucial to building a social climate where people adopt the measures recommended by the authorities [13, 15]. Trust in the authorities will lead people to interpret information appropriately [16, 17] and foster a positive attitude and a collaborative environment that encourages the adoption of protective behaviors [3]. Suppose people do not trust the institutions in charge of protecting them. In that case, people can ignore the information provided by these institutions and also behave oppositely to the behaviors being fostered [17].

Globally, young adults have been identified as the group with the lowest rates of compliance with measures to prevent the spread of the SARS-CoV-2 virus [18, 19], possibly explained by their low risk of severe symptoms [20], the effect of confinement, and physical distancing measures on their mental health [21, 22], or the degree of their distrust in the authorities [19]. Nivette et al. [19] have concluded that non-compliance with COVID-19 protective measures by adolescents and young adults in Zurich, Switzerland, is related to their modest trust in authorities. In addition, within an environment of high distrust in authorities, people can adopt behaviors based on incorrect information about protecting themselves from the virus. For example, they may ingest bleach to prevent infection, inhale alcohol to eliminate the virus or use ultraviolet light on their skin, among other behaviors that can seriously damage their health [23, 24]. Therefore, trust in authorities plays a crucial role in promoting behaviors that protect people’s health within this pandemic.

In March 2020, the first confirmed case of COVID-19 in Chile was detected, which brought the global pandemic to this country. The pandemic arrived in Chile during a significant socio-political crisis. Protests against the government and state authorities in which many people participated, regarding income inequality, the high cost of living, among other demands. The tension increased, and severe episodes of violence and human rights violations occurred, leading the authorities to declare a state of emergency for the first time since the end of the dictatorship [25]. In addition, the government was registering its lowest public confidence indicators. The Organization for Economic Co-operation and Development (OECD) reveals that Chile has the lowest public trust among all OECD countries. Only 15.3% and 17.1% of its citizens trusted the government in 2019 and 2020, respectively [26]. Given the current scenario of a global health crisis and low government trust, the purpose of this study was to determine the influence of government trust on the adoption of health behaviors by young adults to prevent SARS-CoV-2 virus infections in a very unfavorable environment.

### Government Trust and Protective Behaviors

Government trust can broaden people’s knowledge of a pandemic and increase their risk awareness [27]. Since the influenza A (H1N1) pandemic of 2009 and 2010, different studies have shown that high government trust is significantly associated with greater acceptance of protective measures [27–32]. Research by Van der Weerd et al. [30] focused on identifying the factors associated with the intention to adopt protective measures during the influenza A (H1N1) pandemic in the Netherlands. The authors concluded that the highest intent to receive the vaccine was associated with higher government trust, greater fear and worry, and greater perceived vulnerability. Researchers reported similar results in studies related to the Middle East Respiratory Syndrome (MERS) epidemic in early 2012 [33, 34].

During the pandemic caused by the SARS-CoV-2 virus, several studies have identified that trust in authorities is a significant predictor of the acceptance of health measures and the adoption of protective behaviors, such as social distancing [3, 19, 35–38], wearing face masks [4, 18, 36, 39], reducing mobility (staying at home) [35, 40], and getting vaccinated [41–44]. For example, a study in Colombia showed that when government trust is low, measures to prevent covid-19 are rejected and generate anger and disgust, and then cause the levels of distrust in authorities to increase even more [45].

Trust in the authorities is a complex variable studied by researchers from different disciplines. Trust recognizes the role of personal and cultural values and the influence of psychological, social, and political factors [46, 47]. In this study, we examined trust in government concerning its performance in a crisis, that is, the pandemic, and about two dimensions: integrity and competence. Integrity refers to values of honesty and transparency that people perceive in authorities. On the other hand, competence refers to people’s judgment regarding the authorities’ capacities and efficiency in managing a crisis [48, 49]. During a pandemic health crisis, open, honest, and transparent communication is essential to foster public government trust, which encourages the adoption of protective behaviors to mitigate or reduce the damage [27, 50]. Recent studies on the pandemic caused by the SARS-CoV-2 virus have reaffirmed the importance of open and transparent communication between government institutions and the health authorities to drive the adoption of protective behaviors [14, 51]. Thus, we expect government trust to increase health-protective behaviors in this study and propose the following hypothesis.

**H1:** Government trust will have a direct and positive relationship with the adoption of health-protective behaviors.

### Government Trust, Worry/Fear, and Protective Behaviors

Trust in authorities plays a significant role in shaping perceptions in the face of many natural threats [49]. Government trust influences the adoption of protective measures and helps reduce stress and anxiety [5, 50]. In the context of the SARS-CoV-2 pandemic, studies conducted in the United States [3], Singapore [4], China [51], Germany [52], and Latin America [43, 52] have concluded that government trust can increase people’s perception of risk and, therefore, the adoption of protective measures. Siegrist et al. [13] conducted a study during the peak of confirmed COVID-19 cases during the first wave in Switzerland. They found that the perception of risk, measured as fear and worry about infection and the financial impact of the pandemic, was an essential driver of the acceptance and adoption of the measures recommended by the government. In addition, people with high government trust expressed more worry about contagion than people with low social trust. Similarly, Mongue-Rodriguez et al. [53], found in Peru that when people had greater trust in the information about covid-19 provided by the authorities, the population’s perception of risk and perception of treat increased.

The specialized literature indicates that people evaluate risks in cognitive terms (probability) and through emotions such as worry and fear [53–55]. Worry is a crucial determinant of behavior in the context of health-protective behaviors. For example, a study of vaccination behavior in Latin America identified fear as one of the most influential predictors of the intention to be vaccinated [43, 44]. Similar results were reported in a study associated with the A (H1N1) influenza pandemic conducted in the Netherlands [30], Italy [29], Hong Kong [31], the United States [56], and France [57]. In the SARS-CoV-2 virus pandemic context, high levels of worry and fear were associated with increased adoption of protective behaviors [6, 9]. Hence, we expect that in this study, government trust will increase the level of worry and worry, which will increase the adoption of protective behaviors. We, therefore, propose the following hypotheses:

**H2:** Government trust will have a direct and positive relationship with worry/fear.
**H3:** Worry/fear will have a significant and positive effect on adopting health-protective behaviors.

### Government Trust, Subjective Norms, and Protective Behaviors

Trust can also influence protective behaviors through motivational factors that facilitate their adoption [54]. Some studies during the SARS-CoV-2 pandemic have shown that government trust and subjective social norms are significantly related, suggesting that a high degree of government trust may reflect positive values and beliefs by the public that they will be treated with fairness and integrity [3]. In this study, subjective norms refer to the importance that people give to the opinion of others about the adoption of preventive behavior [55, 56].

Lee and Li [3] found that, among the main motivational factors, subjective norms had the most significant influence on social distancing behavior to prevent the spread of the virus that causes COVID-19. The authors also found that subjective norms play a mediating role in the relationship between trust and social distancing behavior. Masser et al. [5] found that subjective norms significantly predicted the intention to donate blood during the pandemic. Trust in blood collection agencies plays a vital role in predicting subjective norms and, indirectly, behavioral intention. Also, a study in Chile identified that when people perceive that their families and friends comply with preventive behaviors against covid-19, they are more likely to wear a mask, keep their distance between people, and wash their hands for more than 20 s [57].

Similarly, Kim and Tandoc [4] found that government trust was positively and significantly related to subjective norms and with the intention to wear a face mask to prevent infection by SARS-CoV-2. Hence, in this study, we expect that government trust will increase social pressure to adopt protective measures and that these social norms will influence the adoption of health-protective behaviors. Consequently, we propose the following hypotheses:

**H4:** Government trust will have a direct and positive relationship with subjective norms.
**H5:** Subjective norms will have a direct and positive relationship with protective behaviors.

### Subjective Norms and Worry/Fear

The Theory of Planned Behavior (TPB) postulates that the direct predictor of behavior is the intention to perform a behavior, which is explained by three motivational factors: attitudes, subjective norms, and perceived behavioral control [55]. Some authors, however, have criticized the TPB because it ignores the role that emotions play in human behavior [58, 59]. For example, perceived threats may cause worry about the consequences of taking or not an action, and worry may strengthen the intention to act [59]. Schmiege, Bryan and Klein [60] have identified that worry significantly affects the attitudes and subjective norms that comprise the TPB. Thus, more worry reinforces subjective norms associated with adopting behaviors, especially among those who had not previously performed that behavior. Raude et al. [6] have found that subjective norms represent the most crucial predictor of compliance with measures to prevent SARS-CoV-2 infection. Subjective norms are significantly related to worry about contracting the coronavirus. Thus, based on this line of reasoning, worry is expected to be significantly associated with subjective norms (e.g., worry will influence social pressure to adopt health-protective behaviors). We, therefore, propose the following hypothesis:

**H6:** Worry/fear will have a direct and positive relationship with subjective norms.

## Methods

### Materials

We tested the hypotheses using a web-based survey of young adults that evaluated health-protective behaviors in response to the COVID-19 pandemic in Chile. The survey contained three sections. The first section measured motivational factors (government trust, worry/fear, and subjective norms). The second section assessed health-protective behaviors (physical distancing, wearing facemasks, and handwashing), and the third one, included questions regarding socio-demographic factors.

Government trust was measured through six items grouped in two dimensions of trust, defined in previous studies [46, 48]. These dimensions comprised three items for integrity-based trust and three items for competence-based trust. Both scales were validated in Chile by previous studies [61]. Participants were asked to respond using a 5-point Likert scale: 1) strongly disagree, 2) disagree, 3) neither agree nor disagree, 4) agree, and 5) strongly agree (see Table 1). Six items characterized the worry/fear variable, adapted from previous studies focused on understanding and predicting the adoption of health-protective behaviors [62, 63]. Participants rated their level of worry and fear on a 7-point scale from (1) not at all to (7) very much. Subjective norms were evaluated using four items that asked about their motivation to comply with four groups of significant people: family, close friends, peers, and teachers (see Table 1). We adapted these items from previous studies that used the TPB in pandemics [63]. As with the item “government trust,” participants were asked to respond using a 5-point Likert scale from (1) strongly disagree to (5) strongly agree. Finally, seven items associated with health-protective behaviors were used, adapted from previous research during the A (H1N1) pandemic [29, 31]. Participants responded using a 5-point frequency scale: 1) never, 2) rarely, 3) sometimes, 4) almost always, and 5) always (see Table 1).

**TABLE 1:** Mean values and standard deviation for each item of government trust, worry/fear, subjective norms, and health protective behavior (Health-protective behaviors to prevent COVID-19, Chile, 2020).

Item description		All sample (*n* = 1,136)	
Trust in the government^a^ (α = 0.91)		Mean	(SD)
Integrity-based Trust			
I1	I am confident that the government will provide all information that is relevant for the health and safety of the public	2.22	(1.16)
I2	I am confident that the government will engage in ongoing open and transparent communication with the public	2.12	(1.15)
I3	I am confident that the government will act without undue political or private pressure	1.85	(1.02)
Competence-based Trust			(1.15)
C1	I am confident that the government has the necessary competencies to make good decisions	2.14	(1.15)
C2	I am confident that the government has the necessary competencies to resolve potential problems	2.19	(1.14)
C3	I am confident that the government has the necessary competencies to adequately communicate its associated risks	2.29	(1.12)
Worry/Fear^b^ (α = 0.82)			
W1	How afraid are you of catching coronavirus (COVID-19)?	4.36	(2.00)
W2	How afraid are you of infecting someone you love with coronavirus (COVID-19)?	6.09	(1.56)
W3	How afraid are you of infecting others with coronavirus (COVID-19)?	5.59	(1.72)
W4	How worried are you about the financial consequences of the COVID-19 pandemic for you and your family?	5.55	(1.70)
W5	How afraid are you of dying from coronavirus?	4.35	(2.29)
W6	I am worried about how the pandemic will evolve	5.73	(1.53)
Subjective Norms^c^ (α = 0.88)			
SN1	What my *family* thinks motivates me to adopt coronavirus prevention measures (COVID-19)	4.12	(1.09)
SN2	What my *best friends* think motivates me to adopt coronavirus prevention measures (COVID-19)	3.75	(1.17)
SN3	What my *colleagues* think motivates me to adopt coronavirus prevention measures (COVID-19)	3.56	(1.19)
SN4	What my *teacher(s)* think motivates me to adopt coronavirus prevention measures (COVID-19)	3.83	(1.16)
Health Protective Behavior^d^ (*α = 0.68*)			
HB1	I wore a face mask on the street and in closed places (supermarket, pharmacy, etc.).	4.96	(0.26)
HB2	I disinfected purchased products with bleach or disinfectant	4.08	(1.21)
HB3	I washed my hands when I got home with an alcohol-based hand sanitizer or soap and water	4.87	(0.48)
HB4	I changed clothes immediately upon arriving home after going out	3.45	(1.35)
HB5	I washed my hands with an alcohol-based hand sanitizer after sneezing, coughing, or wiping my nose	4.11	(1.12)
HB6	When I left my home, I avoided hugging, shaking hands, or kissing on the cheek when greeting another person	4.67	(0.65)
HB7	When I left my home, I tried to keep at least 1 m of distance from other people	4.68	(0.60)

a Items were rated on a 5-point Likert scale, from (1) strongly disagree to (5) strongly agree.

b Items were rated on a 7-point scale, from (1) nothing to (7) very much.

c Items were rated on a 5-point Likert scale, from (1) strongly disagree to (5) strongly agree.

d Items were rated on a 5-point scale, from (1) never to (5) always.

### Procedure and Participants

The survey was constructed and validated by the research team. Then, using a focus group composed of university students, we tested whether the questions were clear and comprehended. The data collection went from September to December 2020 through an online survey. The questionnaire was sent to undergraduate and graduate students of all academic programs of the Andrés Bello University and Pontificia Universidad Catolica of Chile. All respondents consented to participate voluntarily in the study to answer the survey.

One thousand one hundred thirty-six students completed the survey from the cities of Valparaíso, Concepcion, and Santiago. Participants reported an average of 23.3 years (SD = 9.3 years, between 18 and 50 years old). Of the 167 total sample, 83.5% said they were single, 30.7% were male and, 23.5% declared having a family income between 600.000 and 1 million Chilean pesos (726–1452 USD). It took participants an average of 20 min to complete the survey.

### Data Analysis

To test the six hypotheses proposed for this study, an internal reliability analysis of the scales of government trust, worry/fear, subjective norms, and health-protective behaviors was conducted first. We used IBM SPSS Statistics v.27.0 software to calculate Cronbach’s Alpha Coefficient. Kline [64] suggests Alpha values higher than 0.7 for scales with high internal reliability; he also states that values lower than 0.7 can be allowed if the sample size is large enough to estimate all the model’s parameters.

**FIGURE 1:**
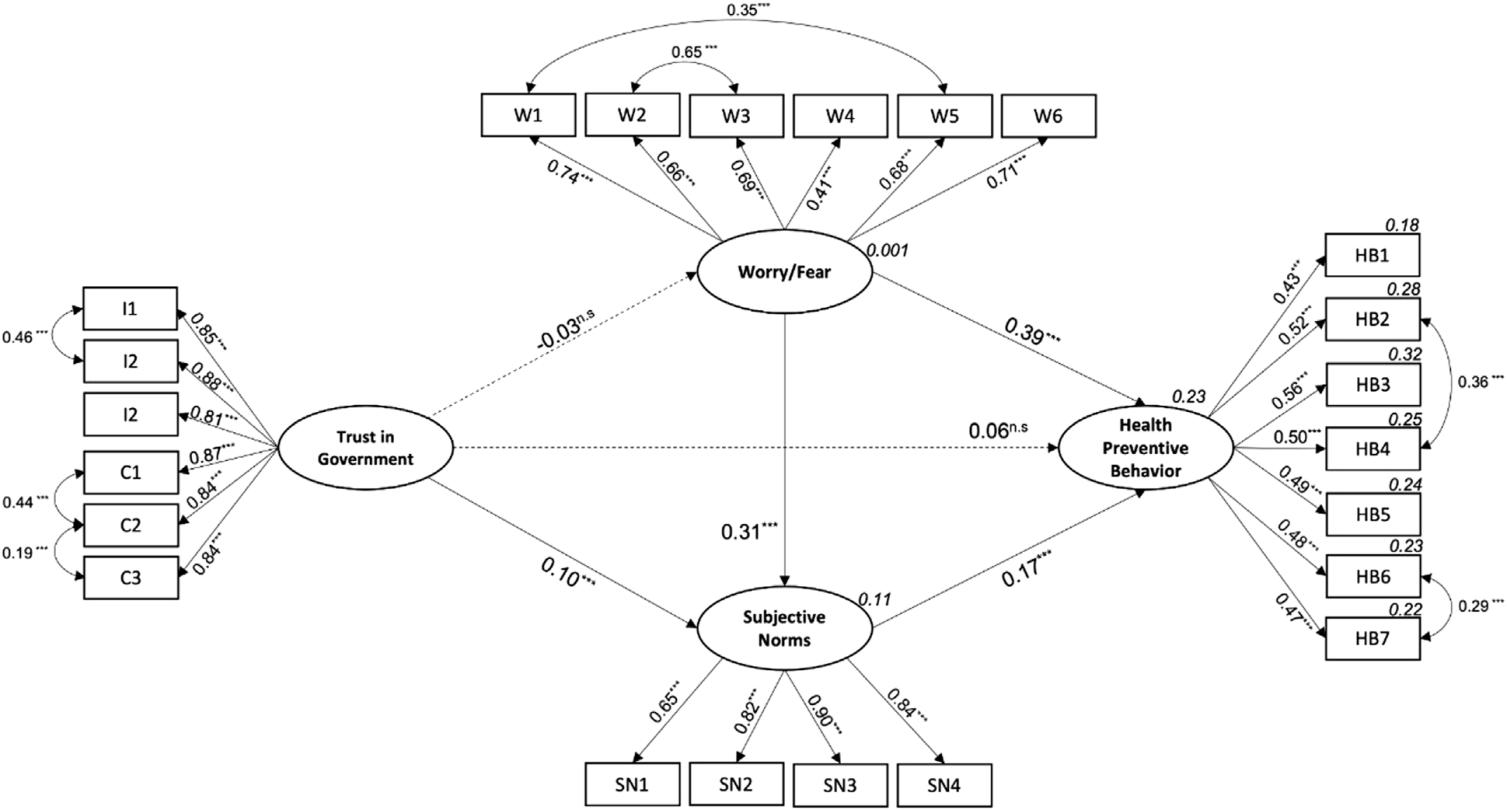
Health Protective Behavior Model (Health-protective behaviors to prevent COVID-19, Chile, 2020). Note: Arrows represent the direct relationships between the latent variables. The values above the arrows indicate the standardized regression coefficients of each relationship. Dotted lines show non-significant standardized regression coefficients. Two-headed arrows show the covariances suggested by the analysis to improve the fit of the model. The values in cursive above the latent variables represent the squared multiple correlations. The cursive values above the health protective observed variables show the proportion of explained variance for each action. Observed variables: Integrity-based Trust (I); Competence-based Trust (T) Worry/Fear (W); Subjective Norms (SN); Health Protective Behaviors (HB). The incorporation of the covariances between Trust in the government items I1-I2, C1-C2, C2-C3 is theoretically justified, since they relate the items within the dimensions of trust studied in this study. The covariances between Worry/Fear items W1–W5 and W2–W3 make theoretical sense, since in the former, both items relate to the fear of direct consequences for the participant, while in the latter, the items relate to the fear of infecting others. Finally, the inclusion of the covariance’s between items HB2-HB4 and HB6-HB7 is reasonable since they relate to health protective behaviors when leaving the home. ***p < 0.001; n.s. non-significant (*p* > 0.05).

IBM SPSS AMOS v.27.0 software was used to build the structural equation model to test the hypotheses of this study. Each latent variable in the model was built using the items associated with each construct as observed variables (see Figure 1). There are no missing values for the observed variables of government trust, worry/fear, and subjective norms. The number of missing values in the health-protective behavior variable was negligible, with 0.9% being the highest percentage found in the sample (item HB5). Given the low number of missing values, we used a non-stochastic imputation method, and the mean replaced the missing values. Maximum likelihood was used to estimate the model, and the following indicators were considered to determine the model fit: chi-squared (χ2), comparative fit index (CFI), normed fit index (NFI), and robustness of mean square error approximation (RMSEA). The proportion of the variance explained by the model was measured using the Squared Multiple Correlation (R^2^_SMC_).

## Results

Table 1 contains the results of the internal reliability analysis, in addition to the mean and standard deviation for each observed variable included in the structural equation model. The Cronbach’s Alpha Coefficients for the four latent variables indicate highly consistent scales.

An analysis of the mean values for the model’s observable variables (see Table 1) indicates that government trust was substantially low, with mean values below 2.3 on a 5-point scale, for all six items. The mean values for worry/fear were moderately high, especially for items associated with fear of infecting loved ones (W2) and uncertainty about how this pandemic will evolve (W6). In terms of subjective norms, participants reported a greater motivation to adopt protective measures when recommended to do so by family members (SN1) and college professors (SN4). Finally, the analysis for the results for health-protective behaviors revealed that participants reported a high frequency in the use of face masks (HB1), hand washing (HB3), and physical distancing (HB6 and HB7).

Table 2 contains the results of the structural equation model fit. Initially, the model did not have a good fit. An analysis of modification indices suggested incorporating seven covariance’s: three for government trust, two for worry/fear, and two for health-protective behavior. The model fit significantly increased by incorporating this theoretically supported covariance (see Figure 1 for details on the covariance). The final model is represented in Figure 1. The analysis confirmed four of the six proposed hypotheses: the relationship between government trust and worry/fear or health-protective behavior was not statistically significant. Hypotheses H1 and H2 were rejected. An analysis of the standardized direct, indirect, and total effects (see Table 3) revealed that worry/fear was the variable that exerted the most significant influence on health-protective behavior (0.44), followed by subjective norms (0.17).

**TABLE 2:** Fit indexes for the structural model (Health-protective behaviors to prevent COVID-19, Chile, 2020).

	χ^2^	*df*	χ^2^/*df*	CFI^a^	NFI^a^	RMSEA^b^	CI90%_RMSEA_
Initial Model (*n* = 1,136)	1,265.27	164	7.72	0.89	0.87	0.08	[0.07–0.08]
Final Model (*n* = 1,136)	475.78	159	2.99	0.97	0.95	0.04	[0.04–0.05]

a According to Hair et al. (2010), values of the Normed Fit Index (NFI) and the Comparative Fit Index (CFI) greater than 0.90 indicate a good model fit, while values greater than 0.95 represent an excellent model fit.

b The Root Mean Square Error of Approximation (RMSEA) must be greater than 0.07 for the model to have a good fit [66].

**TABLE 3:** Standardized direct, indirect, and total effects of the latent variables on health protective behavior (Health-protective behaviors to prevent COVID-19, Chile, 2020).

Latent variables	Direct effect	Indirect effect	Total effect
Trust in Government	0.06	0.01	0.07
Worry/Fear	0.39	0.05	0.44
Subjective Norms	0.17	—	0.17

As measured by the Squared Multiple Correlation (R2SMC) for health-protective behavior, the proportion of variance explained by the model was 23%. A balanced predictive power associated with the protective behaviors included in the study was obtained, ranging between 18% and 32% of the variance explained.

## Discussion

The purpose of this study was to determine the influence of government trust on the adoption of health-protective behaviors by young adults to prevent the effects of the SARS-CoV-2 virus. Based on previous studies, government trust was hypothesized to be directly and indirectly (through worry/fear and subjective norms) related to adopting protective behaviors. This research indicates that worry/fear was the main motivational factor for adopting protective behaviors by young adults, followed by subjective norms. Government trust had only an indirect effect on these behaviors.

### Government Trust

Abundant research reveals that government trust is an essential predictor of accepting and adopting behaviors that prevent the effects and spread of the SARS-CoV-2 virus [19, 35, 37–39]. Government trust during a pandemic implies the belief that information provided by the government is honest and transparent and not influenced by political or private interests [13]. This belief improves communication between the government and the public, allowing for a greater flow of information to protect health [3, 53]. However, our results indicate that government trust did not directly affect preventive behaviors; it only had an indirect influence through subjective social norms on the adoption of protective behaviors by young adults. This relevant result can be explained by the low level of government trust stated by the participants, which is in line with the OECD’s 2019 and 2020 reports on Government trust, The results of this research have important implications for the persons responsible for designing communications strategies in a crisis, such as the pandemic caused by the SARS-CoV-2 virus. The low government trust reported by the participants is an alarming signal for current and future national authorities. In a context of high distrust in the authorities, people seek other reliable actors to obtain information and maintain their capacity to act in a complex environment [58]. In addition, low trust in the authorities can cause more discomfort in the population rejecting the implemented measures and may expose them to adopting erroneous and potentially harmful behaviors in terms of their health based on false or unsupported information by science [45]. Even, as Urrunaga-Pastor et al. [43] pointed out, in a study with a population in Latin America, low trust can influence people’s intention to get vaccinated against COVID, affecting one of the main strategies mitigate the impacts of the pandemic in the region. Therefore, the authorities need to design effective communication strategies that transmit information and increase public confidence, especially in countries with low trust in government.

Contrary to expectations, government trust was not related to fear and worry over the potential financial and health effects of the SARS-CoV-2 pandemic. Siegrist et al. [13] found that fear and worry (the perception of risk) concerning infection and the financial impact of the pandemic were essential drivers for the acceptance and adoption of the measures recommended by the Government in Switzerland. This discrepant result can be explained by the low government trust reported in this study. In relative terms, while the Siegrist et al. [13] study obtained a mean value of 5.74 for social trust on a 7-point scale, our study got a mean value of 2.22 on a 5-point scale.

### Worry/Fear

In the context of health behaviors, worry and fear are essential determinants for people to adopt protective behaviors [9], as they motivate people to follow and implement the measures recommended by the authorities [65]. Our results indicate that worry was the primary motivating factor for adopting health behaviors that prevent the infection and spread of the SARS-CoV-2 virus. This finding is consistent with the results reported by previous research associated with the influenza A (H1N1) pandemic in Italy, Netherlands, and Hong Kong, which reveal that worry was one of the most influential predictors of the adoption of protective measures [29–31]. Therefore, we conclude that people with greater fear and worry report adopting protective behaviors and social distancing more frequently than those who report less fear and worry.

Worry was positively and significantly related to subjective norms, suggesting that fear and worry can increase the impact of social pressure on behavior, decreasing the likelihood that people will try to behave differently. However, as a significant motivational factor for adopting protective health measures, negative emotions’ role cannot be disregarded. If people become familiar with the disease and the associated risks, their levels of worry and fear may decrease. This situation could reduce the frequency with which they adopt protective behaviors, which would be an adverse scenario in controlling the pandemic.

### Subjective Norms

Finally, in line with prior research [3–5], our results suggest that subjective norms were a significant predictor of health behaviors to prevent the virus that causes COVID-19 and have an essential mediating role in the weak relationship between government trust and health-protective behaviors. Similarly, Lee and Li [3] have found that subjective norms were the most influential determinants of social distancing behavior and significantly mediated the relationship between trust and this behavior. In addition, these results are similar to other studies carried out in Chile, which indicate that the opinion and behaviors of friends and relatives are relevant for young people to carry out preventive behaviors against Covid-19 [57]. Then, our results suggest aspects of the Chilean culture and how social relationships are built that can favor decision-making in uncertain and ambiguous situations.

Since subjective norms are a significant predictor of adopting health-protective behaviors, those responsible for designing and implementing risk communication strategies may consider engaging influential groups to increase social pressure on adopting protective behaviors. Our results suggest that communication through influential groups such as family and university professors could be effective for young university students. Higher education teachers usually have greater access to objective and reliable information about the risk of contracting the disease and the measures required to prevent contagion. Therefore, given the higher levels of trust by young university students that higher education professors enjoy, the latter acquires a vital role in risk communication.

### Conclusion

We can conclude that, in a scenario of low government trust—as it occurs in Chile—other motivational factors, such as worry/fear and subjective norms, emerge as the most influential ones for adopting health-protective behaviors among young adults in the face of the pandemic caused by the SARS-CoV-2 virus. People with higher levels of fear and worry about potential health and financial impacts report adopting protective health and social distancing behaviors more frequently than those with less fear and worry. Similarly, young people report a greater adoption of health-protective behaviors when their families and teachers consider adopting these behaviors important.

In addition, the influence of fear and worry evidence the importance of considering emotions as relevant mechanisms for adopting preventive behaviors. Generally, the different models and theories to understand preventive behaviors, such as the TPB model, do not include the direct impact of emotions on health behaviors and only consider cognitive or social factors. However, our study suggests that emotions play an essential role in decision-making when threats are unknown to the population. Consequently, emotions should be studied as factors underlying people’s attitudes or beliefs and valid processes that influence the behavior.

Accordingly, the design and implementation of health prevention strategies need to focus on communicating information based on scientific evidence and restoring government trust. In addition, there is a need to reinforce these behaviors through young people’s significant influencers to create an environment of social cooperation to improve adherence to the current and future health-protective behaviors required to mitigate the impact of the pandemic. Interestingly, family members and university professors appear to be the most relevant among this population, contrary to the influencers commonly recruited to promote behaviors, such as singers, actors, and athletes. University students may be more influenced by their family members and as a way to protect them and their professors, from whom they may expect to learn how to protect their health.

### Limitations

This study has some limitations that must be recognized. First, the cross-sectional data used do not allow inferring causality. Future research should consider longitudinal data to test causal hypotheses. Second, it can be that the global health crisis scenario, the measures implemented by the government to cope with the pandemic, and the prevailing social and political context in Chile may have had a significant influence on people’s perception, acceptance, and adoption of protective health measures. Consequently, additional studies are required to corroborate our findings. Third, the sample was composed of undergraduate and graduate university students with particular socio-demographic characteristics (age or educational level) that may influence their knowledge and behavior to prevent COVID-19.

In consequence, extending these results to the population may be done carefully. Future research should consider a more representative sample of Chile to generalize the results. Finally, the proposed model could have greater predictive power by including new motivational and psychosocial factors into future research, such as the level of trust in the scientific community, in national and international health institutions and organizations, and in the mass media, all relevant stakeholders in the global fight against the pandemic.
